# The ANANKE relative energy gradient (REG) method to automate IQA analysis over configurational change

**DOI:** 10.1007/s00214-017-2113-z

**Published:** 2017-07-05

**Authors:** Joseph C. R. Thacker, Paul L. A. Popelier

**Affiliations:** 1 Manchester Institute of Biotechnology (MIB), 131 Princess Street, Manchester, M1 7DN UK; 2 School of Chemistry, University of Manchester, Oxford Road, Manchester, M13 9PL UK

**Keywords:** Quantum chemical topology, IQA, QTAIM, Energy partitioning, Water dimer, ANANKE, Pearson correlation

## Abstract

Much chemical insight ultimately comes down to finding out which fragment of a total system behaves like the total system, in terms of an energy profile. A simple example is that of the water dimer, where this system is regarded as held together by a hydrogen bond. The hydrogen bond consists of two atoms (H···O), which energetically behave similarly to the total system (H_2_O)_2_. However, from a quantum mechanical point of view, each atom in the total system interacts with *any other* atom. Thus, the view that the hydrogen bond by itself governs the energetic stability of the water dimer needs rigorous justification. In this work, we propose a method that provides such a justification, in general, but only illustrated on the water dimer here. This method is based on the topological energy partitioning method called interacting quantum atoms (IQA). The method is implemented in the program ANANKE, which calculates correlations between the energy profile of the total system and those of subsystems (or fragments). ANANKE acts on the IQA energy contributions obtained for a sequence of full-system geometries controlled by a coordinate of interest (e.g. the O···H distance in the water dimer). Although applied only for the water dimer in this work, the method is general and able to explain the gauche effect, the torsional barrier in biphenyl, the arrow-pushing scheme of an enzymatic reaction (peptide hydrolysis in the HIV-1 Protease active site), and halogen-alkane nucleophilic substitution (S_N_2) reactions. Those applications will appear elsewhere as separate and elaborated case studies; here we focus on the details of the ANANKE method and its justification, using the water dimer as a concrete case.

**Electronic supplementary material:**

The online version of this article (doi:10.1007/s00214-017-2113-z) contains supplementary material, which is available to authorized users.

## Introduction

Chemistry is keen on explaining the (energetic) behaviour of a system by identifying a subsystem that behaves in the same way as the total system. An example of such a subsystem is the hydrogen bond fragment D-H···A, where *D* and *A* are a donor and acceptor atom, respectively. Over the decades (intramolecular), hydrogen bonding has been successful in explaining (and possibly even predicting) the most stable molecular conformations. As another important example, textbooks explain the stability of DNA base pairs through hydrogen bonding. They state that the guanine–cytosine base pair (complex) is more stable than the adenine–thymine base pair because the former has three hydrogen bonds while the latter has only two. In this case, the frontier atoms involved in the intermolecular hydrogen bonding act as a subsystem. However, closer inspection shows that this rationale is not correct. It has been known (e.g. [1–3]) for more almost three decades that some base pairs, while also exhibiting three hydrogen bonds, can nevertheless be two orders of magnitude less stable than guanine–cytosine (all in chloroform). Clearly, the classical subsystem analysis of hydrogen bonding derails here although textbooks have not caught up with this reality.

In 1990 the so-called secondary interaction hypothesis (SIH) [4] was proposed in order to remedy the situation. This hypothesis sought to rescue the idea that the behaviour of a subsystem (the frontier atoms of both base pair monomers) could still explain the behaviour of the total system (a base pair complex). In the guanine–cytosine base pair pattern, hydrogen bonding involves essentially linear D-H···A interactions. In contrast, SIH invokes cross-like interactions between the frontier atoms, instead of only the hydrogen bond-like interactions between atoms that face each other. SIH then counts the number of electrostatic attractions (between partially negative and positive charges) and the number of electrostatics repulsions (between positive and positive, or negative and negative). The formal sum of the number of attractions and repulsions claims to be able to predict the stability ranking between various complexes. SIH was eagerly seized by the supramolecular community (e.g. [5–10]), which was then (and perhaps still is) in need for back-of-the-envelope guidance on complex stability beyond that given by hydrogen bonding. However, SIH is also known to fail or require further modification [5, 6, 11–13]. Moreover, a critical computational study, published [14] in 2002, showed that SIH is not working for the reasons that it purports (if it ever worked in the first place). The conclusion ever since, at least from our point of view, is that a more powerful and rigorous method needs to be proposed. The current article aims at achieving this and will explain in detail how such a method can be justified, while being illustrated on the water dimer.

It is strategically advisable to root such a method in quantum mechanics and immediately call in an atomic partitioning scheme that is parameter free [15]. An obvious choice in that respect is the quantum theory of atoms in molecules (QTAIM) [16–19]. This approach capitalises on the fact that the electron density of any system (molecule or assembly thereof, or condensed matter) naturally falls apart into space-filling atoms, only by applying the gradient vector to the electron density. All approaches that extract (chemically useful) information from the wave function, using only the gradient vector operating on a corresponding property density, are bundled under the name quantum chemical topology (QCT) [20, 21]. To date, there are at least ten approaches [17] that resort under the umbrella of QCT. The approach that is central to the current work is that of interacting quantum atoms (IQA) [22]. This is a robust topological energy partitioning method that disentangles the total energy of a system into intra- and interatomic contributions of various types. IQA is inspired by earlier work [23] and has been used by several groups to study a wide variety of interactions and phenomena, including but not limited to: halogen–halogen interactions in perhalogenated ethanes [24], halogen bond formation [25], conformational analysis of diheteroaryl ketones and thioketones [26], proton transfer reactions [27], formation of an intramolecular bond path between two electronegative atoms [28], substituent effects in electronically excited states [29], cooperative and anti-cooperative effects in resonance-assisted hydrogen bonds in malondialdehyde [30], short-range electrostatics in torsional potentials [31], new insights in atom–atom interactions for future drug design [32], the anomeric effect in halogenated methanols [33], hydrogen–hydrogen interaction in planar biphenyl [34], the steric repulsion in congested molecules [35], charged hydrogen-bonded complexes [36], trapping of CO_2_ by adduct formation [37], and the diastereoselective allylation of aldehydes [38].

Amongst the various energy decomposition analysis (EDA) schemes in existence, IQA presents itself as a modern alternative offering a growing number of applications. Following the EDA acronym tradition, IQA could be referred to as QCTEDA. Older EDA schemes are derived from either a variational method going back to the 1971 *Ansatz* [39] of Morokuma or the (symmetry adapted) perturbation method explained and reviewed in 1994 by Jeziorski et al. [40]. The original Morokuma scheme posed limitations, but many developments strove to overcome them and provide more chemical significance to its energy components, which are unfortunately not uniquely defined. A very recent review [41] on traditional (i.e. non-IQA) EDA schemes highlights and discusses a number of typical problems. One problem is that, at close intermolecular distances and with large basis sets, the separation of charge transfer and polarisation becomes increasingly ill-defined, and numerical instabilities may occur. The space-filling (i.e. no-overlap and no-gap) nature [42] of the topological atoms, which is at the heart of IQA, makes sure that IQA does not suffer from this difficulty. A problem with the Kitaura–Morokuma variational EDA scheme [43] concerns a remainder energy term, called the “mixing term”. This term describes the contribution to the interaction energy not ascribable to any partitioning component of this scheme and has no physical equivalent. Moreover, the mixing term can turn out to be to greater in magnitude than the total interaction energy itself. Later EDA schemes such as the reduced variational space (RVS) analysis [44], constrained space orbital variation (CSOV) [45], and NEDA scheme [46] have been developed to overcome these limitations. As seen within other schemes, the description of the electrostatic interaction between the monomers, and the exchange repulsion contribution as individual terms remains problematic due to their wavefunction definitions not obeying the Pauli principle. Thirdly, the interpretation of the so-called deformation energy term in NEDA, which is based on natural bond orbital (NBO) [47] analysis, is also somewhat problematic because it includes both the contribution of Pauli repulsion and the intra-atomic (or “self”) energy penalty. Again, IQA does not suffer from this or the previous drawback [48]. Finally, IQA does not suffer from another problem with NBO brought up by Stone [49] who writes “The recognition that the NBO estimate of charge-transfer energy is swamped by BSSE means that it is useless in any discussion of intermolecular interactions”.

From the brief analysis above, it should be clear that IQA offers a promising route to address a fundamental question of chemical interpretation: *which part of a given system behaves in the same way as the total system when this system is geometrically altered?* Quantum mechanically, *all* atoms in a given system interact with *all* other atoms in that system, covering three energy types: electrostatic, exchange, and electron correlation. There is a fourth energy type of physical rather than chemical nature: the kinetic energy. This type only occurs within an atom (“intra”) rather than between atoms (“inter”). An attractive and actually essential feature of QTAIM (and hence QCT) is that a topological atom has a unique and well-defined kinetic energy [50]. In other words, the particular shape of its atomic volume makes this possible; this is not the case for an arbitrary subspace. Returning to the totality of the energy partitioning, one ends up with a rapidly unsurveyable number of energy contributions—certainly not a set from which one could intuitively and immediately extract a chemically relevant subset. However, this paper offers a protocol to do exactly that.

Energetic partitioning schemes are often applied to small molecules, or the analysis involves only studying a small subset of interactions without being exhaustive (due to the large number of energetic terms). It would be useful to have a method by which all possible energetic terms can be studied without personal bias or arbitrary parameters. To this end, we have developed what we call the *relative* *energy* *gradient (REG) method.* Essentially, *this method inspects the relation of each energetically partitioned term to the total energy of the system* and ranks all terms in a manner to be explained below. The top-ranked energy term(s) will then offer the sought chemical explanation for the behaviour of the total system.

This proposed solution is actually straightforward and effective. However, we only arrived at it after some failed attempts. Therefore, it is important to explain the reasoning leading to the solution in great detail and illustrate the final method by means of the water dimer. The REG method has been coded in an in-house program called ANANKE. This computer program has been successfully applied to larger systems than the water dimer, such as biphenyl, the gauche effect in 1,2-difluoroethane, peptide hydrolysis in the HIV-1 protease active site, and NS5B HCV RNA polymerase. Those case studies are so involved that they deserve separate publications, to be submitted in due course. The emphasis of the current paper, however, is on the logical path followed in proposing the REG method.

## Theoretical background: interacting quantum atoms (IQA)

This approach has been reviewed many times (see any of the dozen IQA papers listed in one paragraph in Sect. 1). This is why we curtail IQA’s explanation here to the essential. The IQA approach partitions the total energy of a system into intra-atomic and interatomic contributions, or

1 $$E_{\text{total}} = \sum\limits_{A} {E_{\text{IQA}}^{A} } = \sum\limits_{A} {\left( {E_{\text{intra}}^{A} + \frac{1}{2}\sum\limits_{B \ne A} {V_{\text{inter}}^{AB} } } \right)} = \sum\limits_{A} {E_{\text{intra}}^{A} } + \frac{1}{2}\sum\limits_{A} {\sum\limits_{B \ne A} {V_{\text{inter}}^{AB} } } ,$$

where *A* and *B* represent all atoms in the system. The total energy of the system is recovered when all energetic components are summed. Note that $E_{\text{IQA}}^{A}$ is the energy of a given atom *A*, combining its internal energy with the interaction energy between it and its complete atomic environment. The intra-atomic energy can be further partitioned as

2 $$E_{\text{intra}}^{A} = T^{A} + V_{\text{ee}}^{AA} + V_{\text{en}}^{AA} ,$$

where *T* ^*A*^ represents the kinetic energy of the electrons in atomic basin *A,* $V_{\text{ee}}^{AA}$ the intra-atomic electron–electron potential energy, and $V_{\text{en}}^{AA}$ the intra-atomic electron–nuclear potential energy. The interatomic energy can also be further partitioned,

3 $$V_{\text{inter}}^{AB} = \left( {V_{\text{nn}}^{AB} + V_{\text{en}}^{AB} + V_{\text{ne}}^{AB} } \right) + V_{\text{ee}}^{AB} .$$

Note that $V_{\text{en}}^{AB} \ne V_{\text{ne}}^{AB}$ in Eq. 3 because the subscript “en” represents the electron density of atom *A* interacting with the nucleus of atom *B*, and the subscript “ne’” represents the nucleus of atom *A* interacting with the electron density of atom *B*. The energy quantity $V_{\text{nn}}^{AB}$ represents the internuclear repulsion between the nuclei of atoms *A* and *B,* while $V_{\text{ee}}^{AB}$ represents the interatomic electron–electron potential energy. The $V_{\text{ee}}^{AB}$ energy term can be further partitioned as

4 $$V_{\text{ee}}^{AB} = V_{\text{coul}}^{AB} + V_{x}^{AB} + V_{\text{corr}}^{AB} ,$$

where $V_{\text{coul}}^{AB}$ represents the Coulombic energy between the electrons in atoms *A* and *B*, $V_{x}^{AB}$ represents the exchange interaction between the electrons in atoms *A* and *B*, and $V_{\text{corr}}^{AB}$ represents the electron–correlation energy between the atoms *A* and *B*. We note that computational schemes that go beyond Hartree–Fock and introduce correlation lead to the definition of exchange–correlation energy, denoted $V_{xc}^{AB}$, which is the sum of the exchange and correlation energies. The quantity $V_{xc}^{AB}$ can be calculated, within the context of density functional theory (in particular B3LYP), by two possible and alternative *Ansätze*, both recently published [51, 52]. We note that in 2016, MPn-IQA (*n* = 2, 3 or 4) also became possible [53] but this route is still prohibitively expensive for systems larger than the water pentamer.It is possible to redefine the interatomic energy as the sum of two contributions, that is,

5 $$V_{\text{inter}}^{AB} = V_{\text{cl}}^{AB} + V_{\text{xc}}^{AB} .$$

Using Eqs. 3 and 4, it is easy to show that the “classical” electrostatic term ($V_{\text{cl}}^{AB}$) is defined as in Eq. 6,

6 $$V_{\text{cl}}^{AB} = \left( {V_{\text{nn}}^{AB} + V_{\text{en}}^{AB} + V_{\text{ne}}^{AB} } \right) + V_{\text{coul}}^{AB} .$$

The exchange–correlation energy is generally considered to correlate with the covalent interactions between the two atoms.

## Computational details and test system

**Fig. 1:**
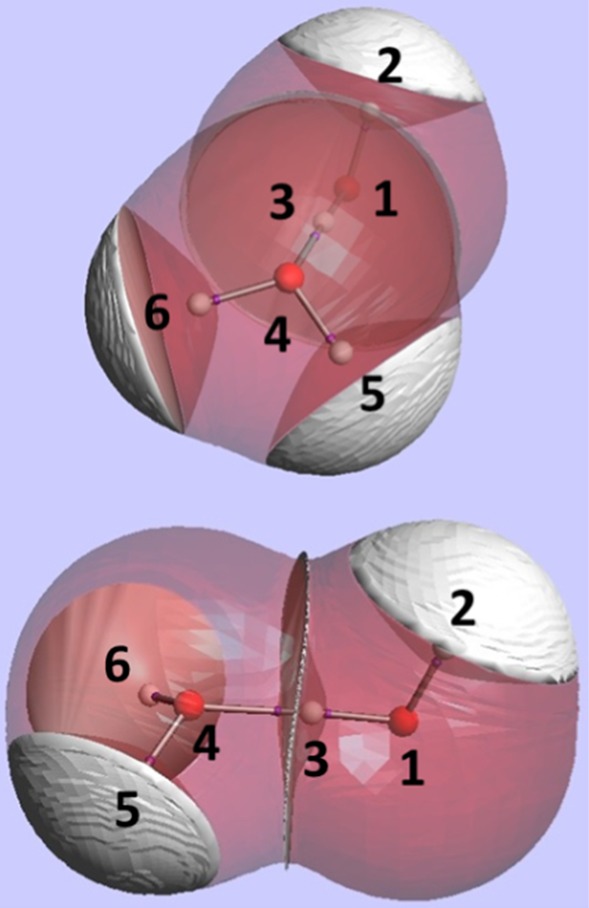
Two views of the same configuration of the water dimer, with atoms marked by numerical labels used throughout this article. The relative orientation of the monomers is the same as that of the global energy minimum, which contains a mirror plane and a single hydrogen bond. Topological atoms are bounded by interatomic surfaces at the system’s inside and by envelopes of constant electron density at the outside. The bond critical points (which are at the respective centres of the interatomic surfaces) are marked by *small purple balls*. These images were generated by the in-house program [56] IRIS, which is based on a finite element algorithm [57]

Figure 1 shows two views of the configuration of the water dimer, which is known to correspond to the global energy minimum. The water dimer is used as a case study to explain and illustrate the REG method. The coordinate in control of the geometric change of the system is the distance between O_4_ and H_3_, which is the length of the hydrogen bond itself. This distance coordinate was varied within the range of 1.25–3.25 Å, with a step size of 0.25 Å, leading to 10 molecular configurations. Starting from the global energy minimum the hydrogen bond length was varied and then fixed for each of the 10 molecular configurations, while all other degrees of freedom were allowed to relax while enforcing a *C*_s_ point group. All geometry optimisation calculations were carried out using GAUSSIAN09 [54] at B3LYP/aug-cc-pVTZ level. All IQA calculations were performed using the AIMAll (Version 16.01.09) package [55].

The REG analysis was performed by the in-house program ANANKE on all pairwise *V*_*cl*_ and *V*_*xc*_ terms and on intra-atomic energy contributions (*E*_intra_). The water dimer system has been studied before, using IQA, by Martín Pendás et al. [58]. They concluded that the hydrogen bond can be described well by the exchange energy. However, they also stated that, due to the complicated nature of the problem of energy decomposition, there are multiple possible interpretations of the IQA data. Already such a simple system is not straightforward in terms of establishing a link between the quantum mechanics that governs it and the chemical view of it. We also remark that in their study the authors only looked at the water dimer at its global energy minimum (alongside eight other hydrogen-bonded complexes also involving HF and NH_3_). It is important to explain the difference between the results of the current paper and those of Martín Pendás et al. Their analysis correlated each IQA term involved in the hydrogen bond of each of the eight complexes to the binding energy of the respective complex. They found that the *V*_x_ (H···A) energy (where *A* is the hydrogen bond acceptor) best correlated to the binding energy. In contrast, in the current study we look at *a number of geometries of a single dimer (i.e. the water dimer)* as the distance between the two monomers is varied. This variation in geometry is crucial for the REG method to be able to operate.

## The relative energy gradient (REG) method

### Overall goal

The REG method analyses potential energy surfaces (PES) arising from an energetic partitioning. The method has two primary objectives:

(i) to *determine the subset* of partitioned energies that best describes the behaviour of the total energy of the system when any coordinate is changed.
(ii) to *extract chemical insight* from an energetically partitioned system.

We will first analyse the problem intuitively and then formulate it mathematically such that it can be implemented in a computer code.

### Preliminary considerations

#### The combinatorial explosion

To focus our thoughts, we work with the water dimer. In this small 6-atom system, there are already 36 energy contributions, all to be included in an exhaustive and automated analysis. These 36(=15 + 15 + 6) energy terms consist of 6 × (6 − 1)/2 = 15 interatomic electrostatic energy terms (*V*_cl_), 15 *V*_xc_ terms and 6 intra-atomic energy terms. From Eq. 1 it follows that these 36 terms add up to the total energy of the system (i.e. the water dimer). Now we vary the geometry of the system via the *control coordinates*, which is the O_4···_H_3_ distance. This coordinate creates an energy profile. The question is now which of these 36 energy terms, or a combination (i.e. sum) thereof, has a profile (again due to coordinate *s* changing) that resembles most the profile of the total system.

If we confine the search to sums of two energy terms, then there are 36 × (36 − 1)/2 = 630 possibilities to investigate. This number rapidly rises to 36 × (36 − 1) × (36 − 2)/3! = 7140 possibilities if sums of three energy terms were considered. If up to six energy terms are considered, then there are just under two million possibilities. Such analysis can still easily be carried out by computer, but the binomial coefficients increase to truly astronomical integer quantities as the number of allowed energy terms increases. Indeed, the maximum value is reached when the number of energy terms is half the total number, that is, 18 = 36/2 in this case. Even if a computer could analyse all the possibilities arising in this combinatorial explosion, then this set would include many possibilities that are chemically meaningless. For example, what would be the chemical meaning of the following three energy contributions behaving like the total system: (i) the intra-atomic energy of a terminal hydrogen (e.g. H_6_), (ii) the exchange energy between the oxygens, and (iii) the electrostatic energy between H_2_ and O_1_ (i.e. the hydrogen bond itself)? Clearly, the construction of a meaningful subset of energy terms is a challenge.

A purely combinatorial strategy could also derail because of degeneracy in the possible answers. How to choose the best answer? Within the spirit of a Pareto front analysis one could consider two competing objectives:

(i) increasing the similarity between the total energy profile and the fragment energy profile (the question of how to define this similarity will be discussed below).
(ii) reducing the number of energy terms forming the subset.

In connection with the second objective, we remark that many small energy contributions can add up to a sizeable contribution but the latter is not desirable or relevant compared to a single much bigger contributor, which “calls the shots”. This effect can already be seen in the water dimer, for example, where $V_{\text{cl}}^{{{\text{H}}3,{\text{O}}4}}$ is a single and dominant energy contribution. Surely, this contribution is chemically more valuable in properly capturing the behaviour of the total energy, compared with 10 small and/or unconnected contributions that might do the same, when added.

In summary, it should be clear from these initial considerations and experimentation that quasi-random and far-fetched combinations of energy terms are as undesirable as having to wait a week or so before a computer returns an answer.

#### The misleading nature of energy differences

**Fig. 2:**
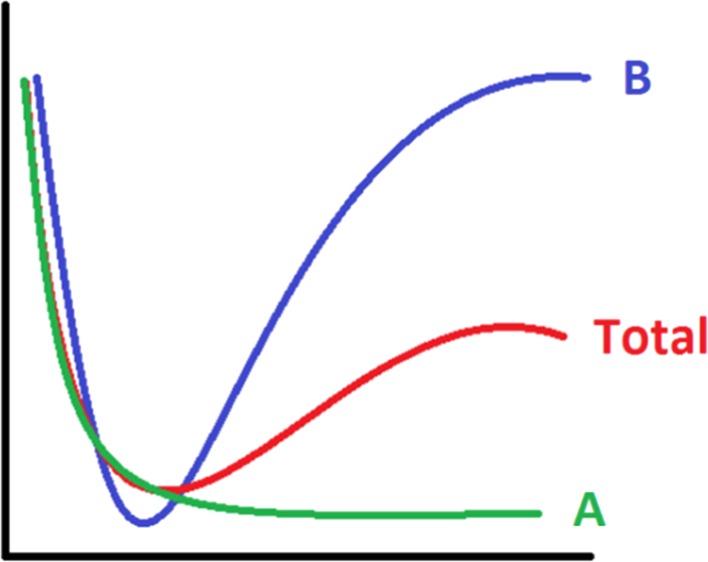
Energy profiles of two candidate energy contributions, *A* and *B*, each of which strives to “mimic” the total energy profile as best as possible. It is observed that RMSE(*A*, total) ≪ RMSE(*B*, total). However, the profile of *B* captures better the overall (broad) behaviour of the total curve in that both *B* and the total energy are wells, while *A* is not

Another natural but actually naïve way to try to reach the overall goal (stated in Sect. 4.1) is measuring the difference in energy between a candidate energy contribution and the total energy. This difference should gauge the *overall* degree of energy difference, rather than at a point-by-point basis. A typical measure is the root-mean-square error (RMSE), calculated between two energy profiles. Figure 2 illustrates the conceivable pitfall when using the RMSE. There are two candidate energy contributions, *A* and *B*, each of which strives to “mimic” the total energy profile as best as possible. Each energy contribution represents a type of energy (e.g. electrostatic, exchange) calculated for a *subset* of atoms. The lower the value of the RMSE, the better the mimicking. One can easily see in Fig. 2 that the RMSE between *A*’s energy profile and the total energy profile is much smaller than the RMSE between *B*’s energy profile and the total energy profile. This conclusion follows from the fact that *A* perfectly follows the total energy for about half of its trajectory and then deviates less from the total than *B* does. However, *A*’s profile misses the overall shape of the total energy, which is that of a (single) well. In contrast, *B* grasps this shape. Yet, the RMSE does not reward it for this success. In summary, the RMSE can be misleading in judging which energy contribution best captures the behaviour of the total energy.

The discussion of the preliminary considerations puts us in a stronger position to tease out the essential features that the REG method should have in order to work. The following subsection zooms in on the *nature* of the overall goal at hand.

### Conceptual background

#### Energy barriers

It can be said that the shape (stationary points and barriers) of a PES is where the “chemistry of a system is exhibited”. For example, in order to understand the dihedral rotation within a hydrocarbon, one actually studies the *energy barrier* associated with dihedral rotation [59]. Similarly, in order to understand the mechanism of a reaction, the transition state barrier with respect to both the reactants and products is studied. One’s understanding of chemical dynamics is based upon the relative energetic stability of minima in PESs compared to the energetic instability of maxima. In other words, understanding the dynamics of a chemical system calls for an understanding of the energy gradients acting within that system at a given point on the PES.

**Fig. 3:**
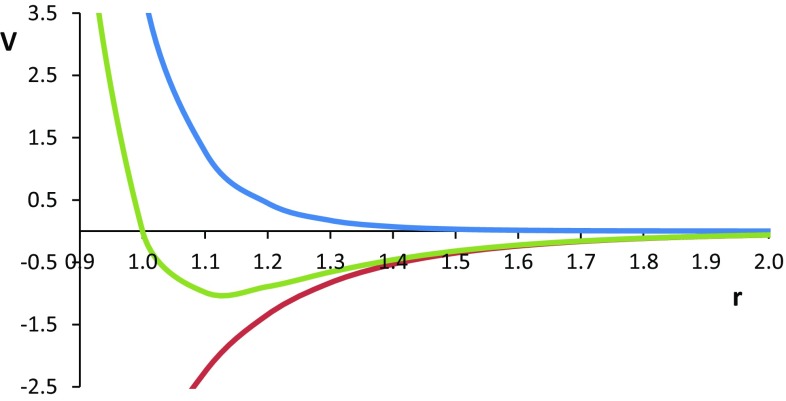
A Lennard-Jones potential (*green*) where both *ε* and *σ* are set to 1 (see Eq. 7), consisting of a repulsive *r* ^−12^ contribution (*blue*) and an attractive *r* ^−6^ contribution (*red*)

Let us now consider the Lennard-Jones potential [60], which consists of two terms, which are very different in nature and both given in Eq. 7,

7 $$V_{LJ} = 4\varepsilon \left[ {\left( {\frac{\sigma }{r}} \right)^{12} - \left( {\frac{\sigma }{r}} \right)^{6} } \right],$$

where *ε* is the depth of the potential well, *σ* is the (finite) distance at which the interparticle potential is zero, and *r* is the distance between the particles. The first (energy) term represents the Pauli repulsion and dominates at short range. The second term represents the dispersion interaction, which dominates at long range. Figure 3 shows a typical energy profile, which consists of two “segments”, one left of the energy minimum and one right of it. It is perhaps more in the spirit of a PES to replace the word “segment” by *barrier*. Indeed, from the point of view of the energy minimum there is a barrier to the left and another barrier to the right. More precisely, there is a monotonic (i.e. if *x* < *y* then *f*(*x*) < *f*(*y*) increase in energy, at both the left and the right of the minimum.

It is visually clear that the *r* ^−6^ (dispersion, red) term dominates the right energy barrier (i.e. long range) because of the confluence of this energy contribution and the total energy. Equally, the left barrier (at short range) is dominated by the *r* ^−12^ (repulsion, blue) term because the gap between this term and the total energy is narrowing. We propose that the principle that a given energy barrier is dominated by one energy contribution, while another barrier by another energy contribution is universal. In other words, as part of the REG method, we postulate that the energy barriers on a PES are caused by different energy contributions.

We note that, by altering the investigated window of the control coordinate or increasing its resolution (i.e. step size) within a single barrier, it is possible to reveal new local regimes, where a type of IQA energy contribution prevails. For example, one can imagine increasingly dominant attractive interactions between large anions due to exchange (rather than electrostatics), occurring at short range, if so probed and indeed revealed.

In summary, when understanding the REG method, it is important to recognise that each of the energy barriers (or wells) on a PES is *caused by a different energy contribution.* As each barrier is caused by a different type of interaction, the PES must be segmented according to its stationary points (i.e. where the gradient vanishes, thus including minima, maxima, and saddle points).

#### Correlation curves

The analysis of the example of the Lennard-Jones potential discussed the behaviour of an energy term (e.g. dispersion) as a function of the control coordinate *s*, which was an interatomic distance. If we denote this energy term *X*, then we discussed *X* = *f*(*s*). Equally, we discussed the energy profile of the total system *Y* as a function of *s*, or *Y* = *g*(*s*). However, what we are really interested in is not the individual functions *f* and *g* but the way *X* and *Y* behave *relative to each other*. In other words, after the control coordinate has done its work in terms of perturbing the system, we look at the correlation between their responses to this perturbation. The next step is thus to look at *r*(*X*, *Y*), which we take to be the Pearson correlation coefficient (defined below) between two energy variables *X* and *Y.* Now we are interested in plotting *X* versus *Y* directly, without explicit reference to *s*.

**Fig. 4:**
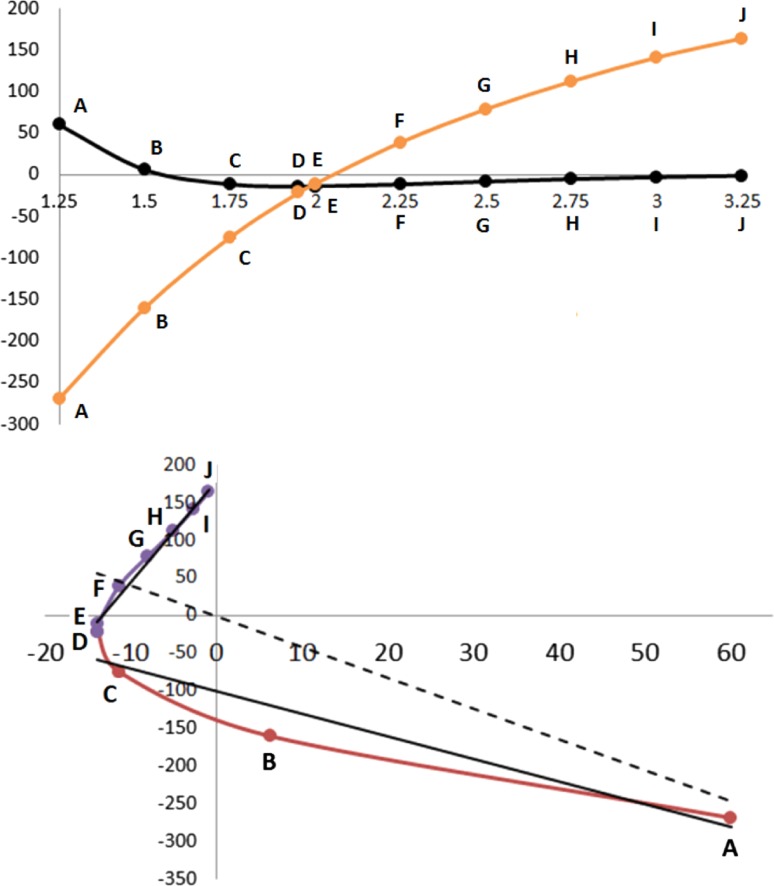
Energy profiles for the water dimer: (*top*) the total energy (*black*) and the *V*_cl_(H3, O4) energy contribution (*orange*) controlled by the O···H distance in Å; (*bottom*) the correlation between the two energies in the *top panel*. The *dashed line* shows the poor linear fit if the whole curve is considered, while much better correlations are obtained after segmentation into a *purple* and a *red curve*

Figure 4 shows a real example, namely that of a water dimer (to be discussed in detail later). The dimer’s geometries are controlled by the H···O distance.

### Mathematical formulation

The REG method is named such due to its derivation through the use of energy gradients. In the following set of equations, the variable *s* stands for the control coordinate, for example, an internuclear distance, dihedral angle, or intrinsic reaction coordinate (IRC). The subscript *i* will denote the energy term, resulting from an energy partitioning, which is IQA in this case study. However, the REG method is more general and does not require that the partitioning method is IQA. The subscript *total* will refer to the total system and hence its behaviour. If the energetic partitioning scheme is additive in nature, which IQA is, then the total energy of the system can be recovered by the following sum,

8 $$E_{\text{total}} (s) = \sum\limits_{i = 1}^{N} {E_{i} (s)} ,$$

where *N* is the total number of energy terms and *s* is the control coordinate, which is sampled at *M* data points. On a practical note, we mention that this equation is numerically not exact for the IQA scheme due to its atomic integration errors [61]. However, these errors are typically controllable to a large extent; in the case of the water dimer, the energy recovery error is <1.1 kJ mol^−1^.

We analyse how the energetically partitioned term changes with respect to the total energy. As explained in Sect. 4.2.2, relating the total energy and an energy term using RMSE can lead to misleading/unreliable results. To overcome this problem, we relate these two energies using linear regression, as shown in Eq. 9.

9 $$E_{i} (s) = m_{{{\text{REG}},i}} \cdot E_{\text{total}} (s) + c_{i} ,$$

where $m_{{{\text{REG}},i}}$ is the REG. Note Eq. 9 actually lists an equation, for every energy term *i*, and fitted to the *M* data points that represent the barrier. It is therefore clear that the REG for a given barrier on a PES can be estimated using the ordinary least squares linear regression equation as shown in Eq. 10,

10 $$m_{{{\text{REG}},i}} = \frac{{\left( {{\mathbf{E}}_{\text{total}}^{\text{translated}} } \right)^{\tau } \cdot {\mathbf{E}}_{i}^{\text{translated}} }}{{\left( {{\mathbf{E}}_{\text{total}}^{\text{translated}} } \right)^{\tau } \cdot {\mathbf{E}}_{\text{total}}^{\text{translated}} }},$$

where

$$\, \begin{array}{*{20}c} {\left( {{\mathbf{E}}_{i}^{\text{translated}} } \right)^{\tau } = \left[ {\begin{array}{*{20}c} {E_{i} (s_{1} ) - \bar{E}_{i} } & {E_{i} (s_{2} ) - \bar{E}_{i} } & \cdots & {E_{i} (s_{M} ) - \bar{E}_{i} } \\ \end{array} } \right]^{\tau } } \\ {\left( {{\mathbf{E}}_{\text{total}}^{\text{translated}} } \right)^{\tau } = \left[ {\begin{array}{*{20}c} {E_{\text{total}} (s_{1} ) - \bar{E}_{\text{total}} } & {E_{\text{total}} (s_{2} ) - \bar{E}_{\text{total}} } & \cdots & {E_{\text{total}} (s_{M} ) - \bar{E}_{\text{total}} } \\ \end{array} } \right]^{\tau } } \\ \end{array}$$

and the superscript bar represents an average over *M* data points, while the translation results from the subtraction of the respective averages.

*The REG is therefore only valid when there is strong linearity between the total energy and the energetically partitioned term.* This is assessed using the Pearson correlation coefficient (*R*_*i*_), as defined in Eq. 11 for the *i*th energy term,

11 $$R_{i} = \frac{{\sum\nolimits_{s}^{M} {(E_{i} (s) - \bar{E}_{i} )(E_{\text{total}} (s) - \bar{E}_{\text{total}} )} }}{{\sqrt {\sum\nolimits_{s}^{M} {(E_{i} (s) - \bar{E}_{i} )^{2} } } \sqrt {\sum\nolimits_{s}^{M} {(E_{\text{total}} (s) - \bar{E}_{\text{total}} )^{2} } } }},$$

where *M* is the number of values of the control coordinate.

The method is called the relative energy gradient method as it actually compares the *gradient* of a given IQA energy term and the gradient of the total system energy. This is clear when the derivative of Eq. 9 is taken with respect to *s*, as seen in Eq. 12,

12 $$\frac{{{\text{d}}E_{i} (s)}}{{{\text{d}}s}} = m_{{{\text{REG}},i}} \cdot \frac{{{\text{d}}E_{\text{total}} (s)}}{{{\text{d}}s}}.$$

Note that Eq. 12 is only valid for perfect correlation and becomes increasingly approximate as the correlation deteriorates. Indeed, in that case, the ratio of derivatives, obtainable from Eq. 12, increasingly deviates from *m*_REG,*i*_. Secondly, we note that, when *s* is a Cartesian coordinate, the REG (i.e. *m*_REG,*i*_) is actually a ratio of forces.

It is important to note that REGs ($m_{{{\text{REG}},i}}$) can have both positive and negative values. Positive REG values represent IQA terms that have the same sign in energy gradient as the total system in the range of the barrier. In other words, the energy gradients associated with these terms have the same sign as the gradient associated with the total system’s energy (i.e. the IQA terms act in the same direction as the barrier). In contrast, negative REG values have an opposite sign to the energy gradient associated with the total energy. Finally, put differently again and concluding, IQA terms with positive (negative) REGs contribute to (de)stabilising the given minimum with respect to a given energy barrier.

A final note concerns a future extension of the REG method. So far it has only been applied, here and in aforementioned publications in preparation, to systems that only vary by one control coordinate (*s*).

Having one single control coordinate often corresponds to a natural choice, such as an IRC, which still governs a set of concerted nuclear displacements. In principle, one could generalise REG to multiple control coordinates but then the question is if the multiple *m*_*REG,i*_ values and Pearson correlation coefficients should be analysed independently, one for each control coordinate, or a single coefficient for the corresponding fitted hyperplane. However, we found that the IRC describing the enzymatic reaction (of the peptide hydrolysis in the HIV-1 protease active site, to be published) sufficed as the only control coordinate needed for a detailed understanding of this reaction.

## Results and discussion

**Fig. 5:**
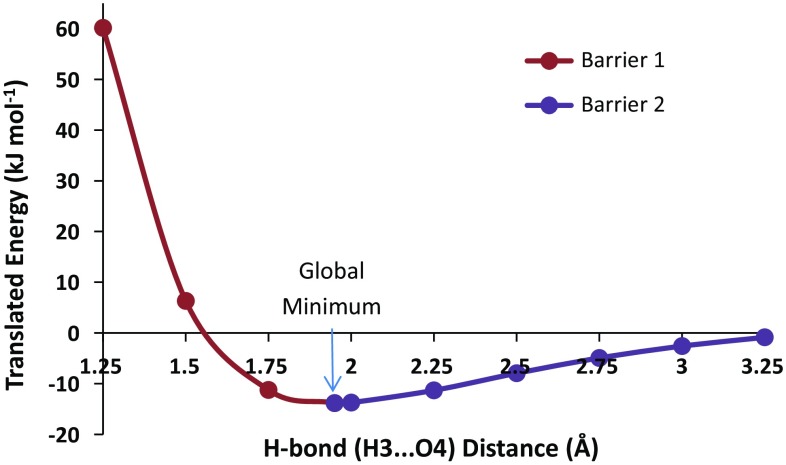
Total energy of the water dimer as the distance coordinate changes. The translated energy is defined as $E_{\text{Total}}^{\text{translated}} (s_{j} ) = E_{\text{Total}} (s_{j} ) - \bar{E}_{\text{Total}} ,$ where *j* = 1, 2, …, *M*. This translation centres the resultant mean energy so that it becomes 0 kJ mol^−1^. Barrier 1 (*left*) consists of four data points, while barrier 2 (*right*) consists of seven data points (as the energy minimum data point is shared by both barriers)

Figure 5 shows the total energy of the water dimer as a function of the distance between the two water molecules, as expressed by the control variable d(H3···O4), where O4 is the hydrogen bond acceptor. The energy minimum is marked by an arrow and appears at ~1.95 Å. From this minimum one can move to the left (i.e. decrease the hydrogen bond length) and experience “(energy) barrier 1”. Similarly, from this same minimum one can move to the right (i.e. increase the hydrogen bond length) and experience “(energy) barrier 2”. These two barriers will be studied independently using REGs. It is clear that barrier 1 represents the repulsive barrier between the two water monomers while barrier 2 represents the attraction between the two monomers.

Table 1 shows that when *R* ≤ 1.95 Å the barrier (i.e. barrier 1) is primarily caused by the *E*_intra_ energy term of the O4 atom (acceptor) because it has the largest REG value, namely 2.1. The intra-atomic energy contribution has recently [62] been linked to interatomic repulsive potentials of the Buckingham type. That work was carried out in the context of intermolecular interactions where complexes of small molecules were systematically compressed in order to investigate the source of their mutual (non-electrostatic) repulsion. The second largest contributor to this barrier is the electrostatic repulsion between the atoms O1 and O4. Chemical intuition would agree with this result because the two large, most negative atoms O1 and O4, repel each other (agreeing with the partial negative charge found on each oxygen atom). The corresponding REG value of 2.0 can be stated in words as that the energy barrier due to the classical electrostatic O1–O4 repulsion occurs at twice the scale of the total energy barrier.

**Table 1:** REGs with the largest absolute magnitude calculated for energy barrier 1 (*R* ≤ 1.95 Å)

Energy terms	REG	Pearson corr. coeff.
E_Intra_o4	2.1	0.98
Vcl_Pair_o1_o4	2.0	0.91
Vxc_Pair_o1_h3	1.7	0.96
Vcl_Pair_h3_h6	1.3	0.96
Vcl_Pair_h3_h5	1.3	0.96
…	…	…
Vcl_Pair_o1_h5	−1.0	−0.93
Vcl_Pair_o1_h6	−1.0	−0.93
Vxc_Pair_h3_o4	−1.5	−0.98
Vcl_Pair_h3_o4	−3.0	−0.95

Entries at the bottom end of Table 1 represent energy terms that very much “work against” the energetic behaviour of the total system, as the distance between the water monomers decreases. The energy term that works against the total energy behaviour to the greatest extent is *V*_cl_(H3, O4), with a REG value of −3.0. This term is the classical electrostatic interaction energy between the hydrogen donor and acceptor. Thus, a term that represents the electrostatic hydrogen bond strengthening (i.e. attraction) is strongly opposing the repulsive energy barrier of the total system. Again, this conclusion agrees with chemical intuition.

Note that the full set of N^2^ = 6^2^ = 36 IQA energy terms are listed in Electronic Supplementary Material (ESM).

**Fig. 6:**
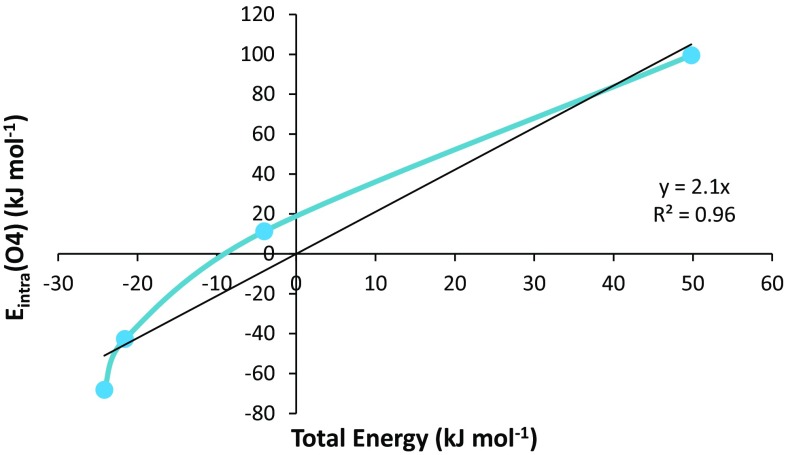
Total energy of the system plotted against the *E*_intra_ of the O4 atom in barrier 1

Figure 6 represents the REG of the *V*_cl_ interaction between O1 and O4 in barrier 1, showing *M* = 4 data points. The REG (i.e. 2.1 in Table 1) for the *V*_cl_ (O1–O4) interaction is the same as the linear gradient given in Fig. 6. Note that Table 1 lists *R* = 0.98, which corresponds to *R* ^2^ = 0.96 in the figure.

It is clear from Fig. 6 that the REG is a linear representation of the gradient of the two energies, which qualitatively represents how the partitioned energy changes, compared to the total energy. It is also clear that there is no explicit control coordinate needed in the calculation of the REG. This is an advantage of the method, as it means that there is no need to rigorously define the control coordinate (which is beneficial when studying an IRC, for example).

Table 2 shows the REG values for the regime of *R* ≥ 1.95 Å associated with barrier 2. It is clear that the term that most contributes to the behaviour of the system is the *V*_cl_ between the atoms H3 and O4. This interaction represents the electrostatic hydrogen bond. It is also clear that this term in the most important as the REG (value of 13.6) is almost twice as large as the two next largest REGs (both 7.1) that belong to the symmetric classical electrostatic interactions O1–H5 and O1–H6. It is also clear that at larger *R* the term that most favours the separation of the two molecules is the classical electrostatic interaction between O1 and O4, or *V*_cl_ (O1–O4). This term, at the bottom of Table 2, has a negative REG value of −15.0. As discussed earlier, such a term very much “works against” the profile of the total energy of the system. Note that this term is the same one featuring as the second largest contributor (Table 1, *R* ≤ 1.95 Å) to the barrier at small *R*.

**Table 2:** REGs with the largest absolute magnitude calculated for barrier 2 (*R* ≥ 1.95 Å)

Terms	REG	Pearson corr.
Vcl_Pair_h3_o4	13.6	0.99
Vcl_Pair_o1_h5	7.1	1.00
Vcl_Pair_o1_h6	7.1	1.00
Vcl_Pair_o1_h3	6.1	0.98
Vcl_Pair_h2_o4	4.7	1.00
Vxc_Pair_h3_o4	2.9	0.96
Vcl_Pair_o4_h5	1.8	0.98
Vcl_Pair_o4_h6	1.8	0.98
…	…	…
Eintra_o4	−3.0	−0.95
Vxc_Pair_o1_h3	−4.0	−0.97
Vcl_Pair_h3_h6	−5.9	−0.99
Vcl_Pair_h3_h5	−5.9	−0.99
Vcl_Pair_o1_o4	−15.0	−1.00

**Fig. 7:**
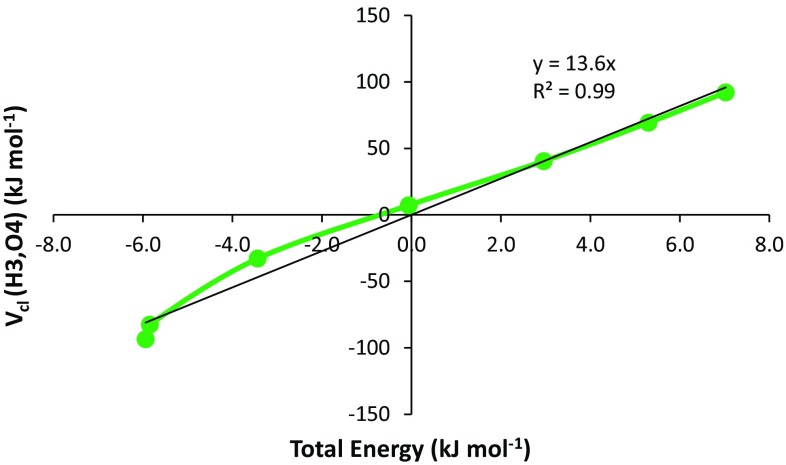
Total energy of the system plotted against the *V*_cl_ of the H3–O4 interaction in barrier 2

Figure 7 shows that the REG can well represent the behaviour of two energies with respect to one another when the Pearson correlation coefficient is close to positive or negative unity. This is why the Pearson correlation coefficient is a crucial metric in this analysis, as it is used to validate the REG method.

**Fig. 8:**
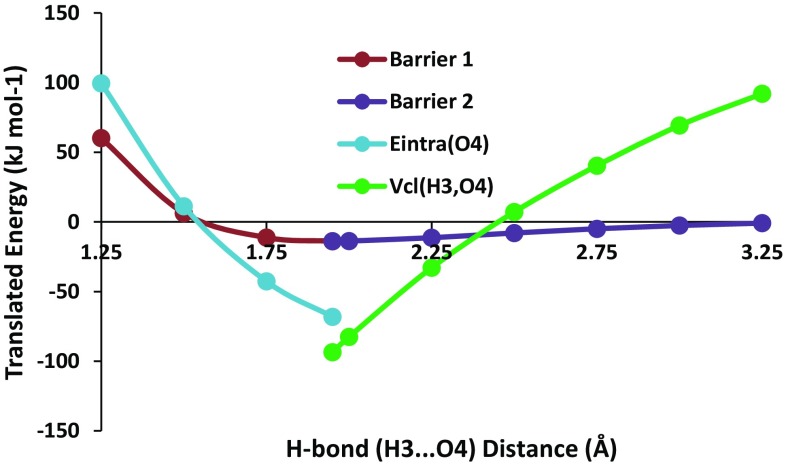
Total energy of the system plotted along with the largest REGs of each barrier

Figure 8 graphically represents the energetic terms that have the largest REGs. Intuitively, it is apparent that the REGs are related to the gradient of the energy term. The largest REGs can be seen to contribute large gradients in the direction of the total energy PES. This is clearly shown in Fig. 8.

It is also possible to go beyond (Fig. 8) and attempt to reconstruct the total energy profile more precisely. Inspection of the reconstruction of barrier 1 calls for an energy term that dampens the *E*_intra(O4) term. In other words, we search for a term that works *against* the barrier that *E*_intra(O4) created. A natural choice is to look for an energy term with a large and negative REG value in barrier 1, because this term has the clearest chemical meaning. As one continues this addition of terms with very positive REG values and very negative REG values, the total energy barrier becomes increasingly better approximated. Of course, in the limit of summing all terms one recovers the total energy barrier exactly. However, one wishes to stay away from this limit because reaching it defeats the original goal of recovering the smallest (and chemically more meaningful) subset of energy terms. Note that the more terms from the middle region are included (i.e. with REG values of small absolute value), the more one introduces possible noise. The latter would dilute the quality of the answer that ANANKE can give. Reconstructing barrier 2 follows the same protocol, that is, by a sum of energy terms that alternate between large positive and large negative REG values.

**Fig. 9:**
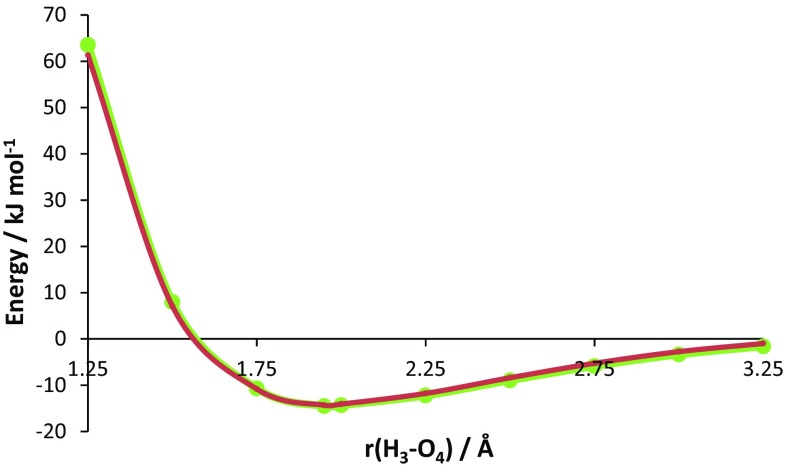
Total energy of the water dimer plotted against the sum of the 28 IQA terms with the largest magnitude REGs. The original unpartitioned energy is marked in *red* while an energy consisting of 28 out 36 possible IQA contributions is marked in *green*

How can one then reconstruct the whole energy profile of the total system from a subset of terms? Naively one could construct each barrier independently, one after the other, by the protocol above. However, our initial goal was to construct the total energy barrier using REG values as our guide. We therefore must also alternate the previous summation over the two barriers. This leads to a protocol where one starts with the IQA term with the most positive REG value in a given barrier, and adds to it the IQA term with most positive REG value in the *other* barrier. To this sum, one then adds the term with the most negative REG value in the first barrier, followed by the term with the most negative REG value in the other barrier. One keeps adding in this manner but now using the *second* most positive or negative REG values, then the third, and so on. After each summation, the RMSE was calculated to monitor the quality of the “subset reconstruction”. In summary, one systematically reconstructs the overall energy barrier by a succession of energy terms of gradually decreasing chemical relevance. As a final note, we point out that no energy terms are summed twice. We applied the protocol to the water dimer, resulting in the superposition of the total energy profile and the reconstructed one, as shown in Fig. 9.

Figure 9 shows that the total energy of the water dimer can be approximated by 28 of a total of 36 REGs. The RMSE between the total energy (E(wavefunction)) and the IQA subset is then only 1.4 kJ mol^−1^.

It is expected that, in systems where chemical change is localised, the percentage of IQA terms required to recreate the total energy of the system will decrease. To this end, it is hoped that this protocol can be used to find only the chemically relevant atoms in, say, the active site of a protein. However, on a related note we can announce that, in currently unpublished work, the REG method has been applied to a number of systems, the largest one of which consists of over 17,000 IQA energy terms. It showed that the energetics of the known reaction mechanism in the active site of a protein can be recovered using the REG method. By considering only the largest pairwise *V*_xc_ interactions in the system an “energetic” arrow-pushing mechanism was obtained to represent bond formation and breaking. Pleasingly, this REG-obtained mechanism reflected the known mechanism but also highlighted additional bonding information showing how the atoms of the protein’s active site participate in catalysis.

## Conclusions

We have presented the REG method in general terms and in detail. This method is able to select energy terms, by both locality and type that energetically behave like the energy of the total system. This is important to extract chemical insight from an energetic partitioning method, which is IQA in this case. We illustrated the method by the water dimer, described at B3LYP level. We also showed that the method can be used to guide the selection of a subset of energy terms to describe semi-qualitatively the behaviour of a system. The method recovers chemical intuition and is a physically meaningful metric that does not rely on a priori knowledge of the system or any arbitrary parameters.

## Electronic supplementary material

Below is the link to the electronic supplementary material.

Supplementary material 1 (DOCX 20 kb)

## References

[1] Hamilton AD, Van Engen D (1987). Induced fit in synthetic receptors: nucleotide base recognition by a “molecular hinge”. J Am Chem Soc.

[2] Kyogoku Y, Lord RC, Rich A (1967). The effect of substituents on the hydrogen bonding of adenine and uracil derivatives. Proc Natl Acad Sci USA.

[3] Kelly TR, Maguire MP (1987). A receptor for the oriented binding of uric acid type molecules. J Am Chem Soc.

[4] Jorgensen WL, Pranata J (1990). Importance of secondary interactions in triply hydrogen bonded complexes: guanine-cytosine vs uracil-2,6-diaminopyridine. J Am Chem Soc.

[5] Murray TJ, Zimmerman SC (1992). New triply hydrogen bonded complexes with highly variable stabilities. J Am Chem Soc.

[6] Jeong KS, Tjivikua T, Muehldorf A, Deslongchamps G, Famulok M, Rebek J (1991). Convergent functional groups. 10. Molecular recognition of neutral substrates. J Am Chem Soc.

[7] Beijer FH, Sijbesma RP, Kooijman H, Spek AL, Meijer EW (1998). Strong dimerization of ureidopyrimidones via quadruple hydrogen bonding. J Am Chem Soc.

[8] Navarro JAR, Freisinger E, Lippert B (2000). From simple trans-[a_2_Pt(2-hydroxypyrimidine)_2_]^2+^ (a = NH_3_, CH_3_NH_2_) complexes to structures of higher complexity. Molecular recognition of 2-aminopyrimidine by hydrogen bond formation and reactivity toward additional metal ions. Inorg Chem.

[9] Lan T, McLaughlin LW (2001). The energetic contribution of a bifurcated hydrogen bond to the binding of DAPI to dA-dT rich sequences of DNA. J Am Chem Soc.

[10] Gardner RR, Gellman SH (1995). Evaluation of the conformation-directing effects of secondary hydrogen-bonding interactions in flexible tetrapeptide analogues. J Am Chem Soc.

[11] Yang J, Gellman SH (1998). Energetic superiority of two-center hydrogen bonding relative to three-center hydrogen bonding in a model system. J Am Chem Soc.

[12] Gardner RR, Gellman SH (1997). Secondary effects in flexible hydrogen bonding networks. Tetrahedron.

[13] Zeng H, Miller RS, Flowers RA, Gong B (2000). A highly stable, six-hydrogen-bonded molecular duplex. J Am Chem Soc.

[14] Popelier PLA, Joubert L (2002). The elusive atomic rationale for DNA base pair stability. J Am Chem Soc.

[15] Popelier PLA, Mingos M (2016). Quantum chemical topology. The chemical bond—100 years old and getting stronger.

[16] Bader RFW (1985). Atoms in molecules. Acc Chem Res.

[17] Popelier PLA (2014) The quantum theory of atoms in molecules. In: Frenking G, Shaik S (eds) The nature of the chemical bond revisited, Chapter 8. Wiley-VCH, Weinheim, pp 271–308

[18] Bader RFW (1990). Atoms in molecules. A quantum theory.

[19] Matta CF, Boyd RJ (2007). The quantum theory of atoms in molecules. From solid state to DNA and drug design.

[20] Popelier PLA, Wales DJ (2005). Quantum chemical topology: on bonds and potentials, structure and bonding. Intermolecular forces and clusters.

[21] Popelier PLA, Banting L, Clark T (2012). Quantum chemical topology: on descriptors, potentials and fragments. Drug design strategies: computational techniques and applications.

[22] Blanco MA, Martín Pendás A, Francisco E (2005). Interacting quantum atoms: a correlated energy decomposition scheme based on the quantum theory of atoms in molecules. J Chem Theor Comput.

[23] Popelier PLA, Kosov DS (2001). Atom–atom partitioning of intramolecular and intermolecular Coulomb energy. J Chem Phys.

[24] Yahia-Ouahmed M, Tognetti V, Joubert L (2015). Halogen–halogen interactions in perhalogenated ethanes: an interacting quantum atoms study. Comput Theor Chem.

[25] Tognetti V, Joubert L, Chauvin R, Lepetit C, Alikhani E, Silvi B (2016). Following halogen bonds formation with Bader’s atoms-in-molecules theory. Challenges and advances in computational chemistry and physics dedicated to “Applications of topological methods in molecular chemistry”.

[26] Matczak P, Domagała M, Domagała S (2016). Conformers of diheteroaryl ketones and thioketones: a quantum chemical study of their properties and fundamental intramolecular energetic effects. Struct Chem.

[27] Inostroza-Rivera R, Yahia-Ouahmed M, Tognetti V, Joubert L, Herrera B, Toro-Labbe A (2015). Atomic decomposition of conceptual DFT descriptors: application to proton transfer reactions. Phys Chem Chem Phys.

[28] Tognetti V, Joubert L (2013). On the physical role of exchange in the formation of an intramolecular bond path between two electronegative atoms. J Chem Phys.

[29] Ferro-Costas D, Francisco E, Martin Pendas A, Mosquera RA (2016). How electronic excitation can be used to inhibit some mechanisms associated to substituent effects. ChemPhysChem.

[30] Romero-Montalvo E, Guevara-Vela JM, Costales A, Martin Pendas A, Rocha-Rinza T (2017). Cooperative and anticooperative effects in resonance assisted hydrogen bonds in merged structures of malondialdehyde. Phys Chem Chem Phys.

[31] Darley MG, Popelier PLA (2008). Role of short-range electrostatics in torsional potentials. J Phys Chem A.

[32] Popelier PLA (2012). New insights in atom–atom interactions for future drug design. Curr Top Med Chem.

[33] Ferro-Costas D, Vila A, Mosquera RA (2013). Anomeric effect in halogenated methanols: a quantum theory of atoms in molecules study. J Phys Chem A.

[34] Eskandari K, Van Alsenoy C (2014). Hydrogen–hydrogen interaction in planar biphenyl: a theoretical study based on the interacting quantum atoms and Hirshfeld atomic energy partitioning methods. J Comput Chem.

[35] Dillen J (2013). Congested molecules. Where is the steric repulsion? An analysis of the electron density by the method of interacting quantum atoms. Int J Quantum Chem.

[36] Alkorta I, Mata I, Molins E, Espinosa E (2016). Charged versus neutral hydrogen-bonded complexes: is there a difference in the nature of the hydrogen bonds?. Chem Eur J.

[37] Alkorta I, Montero-Campillo MM, Elguero J (2017). Trapping CO_2_ by adduct formation with NHC’s: a theoretical study.

[38] Tognetti V, Bouzbouz S, Joubert L (2017). A theoretical study of the diastereoselective allylation of aldehydes with new chiral allylsilanes. J Mol Model.

[39] Morokuma K (1971). Molecular orbital studies of hydrogen bonds. III. C=O···H–O hydrogen bond in H_2_CO···H_2_O and H_2_CO···2H_2_O. J Chem Phys.

[40] Jeziorski B, Moszynski R, Szalewicz K (1994). Perturbation theory approach to intermolecular potential energy surfaces of van der Waals complexes. Chem Rev.

[41] Phipps MJS, Fox T, Tautermann CS, Skylaris C-K (2015). Energy decomposition analysis approaches and their evaluation on prototypical protein–drug interaction patterns. Chem Soc Rev.

[42] Popelier PLA (2016). Molecular simulation by knowledgeable quantum atoms. Phys Scr.

[43] Kitaura K, Morokuma K (1976). A new energy decomposition scheme for molecular interactions within the Hartree-Fock approximation. Int J Quantum Chem.

[44] Chen W, Gordon MS (1996). The effective fragment potential model for solvation: internal rotation in formamide. J Chem Phys.

[45] Bagus PS, Hermann K, Bauschlicher CW (1984). A new analysis of charge transfer and polarization for ligand–metal bonding: model studies of Al_4_CO and Al_4_NH_3_. J Chem Phys.

[46] Glendening EF, Streitwieser A (1994). Natural energy decomposition analysis: an energy partitioning procedure for molecular interactions with application to weak hydrogen bonding, strong ionic, and moderate donor–acceptor interactions. J Chem Phys.

[47] Reed AE, Weinstock RB, Weinhold F (1985). Natural population analysis. J Chem Phys.

[48] Pendas AM, Blanco MA, Francisco E (2007). Chemical fragments in real space: definitions, properties, and energetic decompositions. J Comput Chem.

[49] Stone AJ (2017). Natural bond orbitals and the nature of the hydrogen bond. J Phys Chem A.

[50] Bader RFW, Beddall PM (1972). Virial field relationship for molecular charge distributions and the spatial partitioning of molecular properties. J Chem Phys.

[51] Maxwell P, Martin Pendas A, Popelier PLA (2016). Extension of the interacting quantum atoms (IQA) approach to B3LYP level density functional theory. PhysChemChemPhys.

[52] Francisco E, Casals-Sainz JL, Rocha-Rinza T, Martin-Pendas A (2016). Partitioning the DFT exchange-correlation energy in line with the interacting quantum atoms approach. Theor Chem Acc.

[53] McDonagh JL, Vincent MA, Popelier PLA (2016). Partitioning dynamic electron correlation energy: viewing Møller-Plesset correlation energies through interacting quantum atom (IQA) energy partitioning chem. Phys Lett.

[54] Trucks GW, Schlegel HB, Scuseria GE, Robb MA, Cheeseman JR, Scalmani G, Barone V, Mennucci B, Petersson GA, Nakatsuji H, Caricato M, Li X, Hratchian HP, Izmaylov AF, Bloino J, Zheng G, Sonnenberg JL, Hada M, Ehara M, Toyota K, Fukuda R, Hasegawa J, Ishida M, Nakajima T, Honda Y, Kitao O, Nakai H, Vreven T, Montgomery JA, Peralta JE, Ogliaro F, Bearpark M, Heyd JJ, Brothers E, Kudin KN, Staroverov VN, Kobayashi R, Normand J, Raghavachari K, Rendell A, Burant JC, Iyengar SS, Tomasi J, Cossi M, Rega N, Millam JM, Klene M, Knox JE, Cross JB, Bakken V, Adamo C, Jaramillo J, Gomperts R, Stratmann RE, Yazyev O, Austin AJ, Cammi R, Pomelli C, Ochterski JW, Martin RL, Morokuma K, Zakrzewski VG, Voth GA, Salvador P, Dannenberg JJ, Dapprich S, Daniels AD, Farkas Ö, Foresman JB, Ortiz JV, Cioslowski J, Fox DJ, Gaussian 09 RBMJF (2009). GAUSSIAN09.

[55] Keith TA, AIMAll (Version 14.04.17), (http://aim.tkgristmill.com), T.G.S. Todd A. Keith, Overland Park KS, USA, (aim.tkgristmill.com), Todd A. Keith, TK Gristmill Software, Overland Park KS, USA, (aim.tkgristmill.com), 2014

[56] Rafat M, Devereux M, Popelier PLA (2005). Rendering of quantum topological atoms and bonds. J Mol Graph Model.

[57] Rafat M, Popelier PLA (2007). Visualisation and integration of quantum topological atoms by spatial discretisation into finite elements. J Comput Chem.

[58] Pendás AM, Blanco MA, Francisco E (2006). The nature of the hydrogen bond: a synthesis from the interacting quantum atoms picture. J Chem Phys.

[59] Pophristic V, Goodman L (2001). Hyperconjugation not steric repulsion leads to the staggered structure of ethane. Nature.

[60] Smit B (1992). Phase diagrams of Lennard-Jones fluids. J Chem Phys.

[61] Aicken FM, Popelier PLA (2000). Atomic properties of selected biomolecules. Part 1. The interpretation of atomic integration errors. Can J Chem.

[62] Wilson A, Popelier PLA (2016). Exponential relationships capturing atomistic short-range repulsion from the interacting quantum atoms (IQA) method. J Phys Chem A.

