# Supplementary material for: The ANANKE relative energy gradient (REG) method to automate IQA analysis over configurational change
Source: Theor Chem Acc. 2017 Jul 5;136(7):86. doi: 10.1007/s00214-017-2113-z (PMC6979521; doi:10.1007/s00214-017-2113-z)
Supplement: Supplementary file 1 — Supplementary material 1 (DOCX 20 kb) [file 214_2017_2113_MOESM1_ESM.docx]

**Electronic Supplementary Material**

**The ANANKE relative-energy-gradient method to automate IQA analysis over configurational change**

Joseph C.R. Thacker and Paul L.A. Popelier

Manchester Institute of Biotechnology (MIB), 131 Princess Street, Manchester M1 7DN, Great Britain and

School of Chemistry, University of Manchester, Oxford Road, Manchester M13 9PL, Great Britain

| **Barrier 1** | | |
| --- | --- | --- |
| **Terms** | **Force Ratio** | **Pearson Corr. Coeff.** |
| Eintra_o4 | 2.11 | 0.98 |
| Vcl_o1_o4 | 1.97 | 0.91 |
| Vxc_o1_h3 | 1.68 | 0.96 |
| Vcl_h3_h6 | 1.34 | 0.96 |
| Vcl_h3_h5 | 1.34 | 0.96 |
| Eintra_h3 | 0.63 | 0.89 |
| Eintra_o1 | 0.39 | 0.70 |
| Eintra_h5 | 0.33 | 0.98 |
| Eintra_h6 | 0.33 | 0.98 |
| Vxc_o4_h5 | 0.33 | 0.98 |
| Vxc_o4_h6 | 0.33 | 0.98 |
| Vcl_o1_h2 | 0.29 | 0.99 |
| Vcl_h5_h6 | 0.25 | 0.98 |
| Vcl_h2_h6 | 0.22 | 0.91 |
| Vcl_h2_h5 | 0.22 | 0.91 |
| Vxc_h2_h3 | 0.01 | 0.93 |
| Vxc_h5_h6 | 0.01 | 0.98 |
| Vxc_h2_h5 | 0.00 | -0.99 |
| Vxc_h2_h6 | 0.00 | -0.99 |
| Vxc_h3_h6 | 0.00 | -0.94 |
| Vxc_h3_h5 | 0.00 | -0.94 |
| Vxc_h2_o4 | -0.01 | -0.97 |
| Vxc_o1_h5 | -0.02 | -0.99 |
| Vxc_o1_h6 | -0.02 | -0.98 |
| Vxc_o1_h2 | -0.27 | -0.97 |
| Eintra_h2 | -0.30 | -0.97 |
| Vcl_o1_h3 | -0.31 | -0.36 |
| Vcl_h2_h3 | -0.36 | -0.99 |
| Vcl_h2_o4 | -0.39 | -0.87 |
| Vcl_o4_h6 | -0.84 | -0.98 |
| Vcl_o4_h5 | -0.84 | -0.98 |
| Vxc_o1_o4 | -0.90 | -0.96 |
| Vcl_o1_h5 | -1.02 | -0.93 |
| Vcl_o1_h6 | -1.03 | -0.93 |
| Vxc_h3_o4 | -1.47 | -0.98 |
| Vcl_h3_o4 | -2.99 | -0.95 |

| **Barrier 2** | | |
| --- | --- | --- |
| **Terms** | **Force Ratio** | **Pearson Corr. Coeff.** |
| Vcl_h3_o4 | 13.64 | 0.99 |
| Vcl_o1_h5 | 7.14 | 1.00 |
| Vcl_o1_h6 | 7.07 | 1.00 |
| Vcl_o1_h3 | 6.13 | 0.98 |
| Vcl_h2_o4 | 4.71 | 1.00 |
| Vxc_h3_o4 | 2.88 | 0.96 |
| Vcl_o4_h5 | 1.82 | 0.98 |
| Vcl_o4_h6 | 1.81 | 0.98 |
| Vxc_o1_o4 | 1.76 | 0.96 |
| Eintra_h2 | 0.63 | 0.97 |
| Vxc_o1_h2 | 0.57 | 0.97 |
| Vcl_o1_h2 | 0.39 | 0.99 |
| Vxc_h2_o4 | 0.03 | 0.96 |
| Vxc_h3_h6 | 0.02 | 0.97 |
| Vxc_h3_h5 | 0.02 | 0.97 |
| Vxc_o1_h6 | 0.02 | 0.93 |
| Vxc_o1_h5 | 0.02 | 0.93 |
| Vxc_h2_h6 | 0.00 | 0.93 |
| Vxc_h2_h5 | 0.00 | 0.93 |
| Vxc_h5_h6 | -0.01 | -0.98 |
| Vxc_h2_h3 | -0.03 | -0.98 |
| Vcl_h2_h3 | -0.35 | -1.00 |
| Vcl_h5_h6 | -0.67 | -0.98 |
| Vxc_o4_h5 | -0.75 | -0.98 |
| Vxc_o4_h6 | -0.75 | -0.98 |
| Eintra_h6 | -0.78 | -0.98 |
| Eintra_h5 | -0.78 | -0.98 |
| Vcl_h2_h6 | -2.21 | -1.00 |
| Vcl_h2_h5 | -2.24 | -1.00 |
| Eintra_h3 | -2.59 | -0.97 |
| Eintra_o1 | -2.59 | -0.97 |
| Eintra_o4 | -3.01 | -0.95 |
| Vxc_o1_h3 | -4.04 | -0.97 |
| Vcl_h3_h6 | -5.88 | -0.99 |
| Vcl_h3_h5 | -5.92 | -0.99 |
| Vcl_o1_o4 | -15.03 | -1.00 |
